# Reversible causes of death and the potential benefit of invasive emergency techniques in paediatric and adolescent trauma: a 12-years retrospective forensic analysis

**DOI:** 10.1186/s12873-025-01469-5

**Published:** 2026-01-09

**Authors:** Leila Malolepszy, Stephan Heidl, Melanie Markmann, Thomas S. Zajonz, Niko Schneider, Christian Koch, Sven Hartwig, Michael Sander, Emmanuel Schneck

**Affiliations:** 1 Institute for Forensic Medicine, Justus Liebig University Giessen, University Hospital Giessen and Marburg, 35392 Giessen, Germany; 2 Department of Anesthesiology, Intensive Care Medicine and Pain Therapy, Justus Liebig University of Giessen, University Hospital Giessen and Marburg, Rudolf-Buchheim-Strasse 7, 35392 Giessen, Germany

**Keywords:** Trauma, Cardiac arrest, REBOA, Resuscitative thoracotomy, Prehospital transfusion

## Abstract

**Background:**

Early deaths among severely injured children are mainly due to exsanguination and severe traumatic brain injury, some of which may be preventable with timely emergency interventions. However, data on potentially reversible causes of death and the role of advanced prehospital procedures in paediatric trauma are scarce. This study aims to assess post-mortem findings to identify cases where advanced emergency interventions could have been applicable in children.

**Methods:**

This retrospective, single-centre study analysed forensic files of deceased children and adolescents (age < 18 years) regarding the potential benefit of advanced emergency procedures (e.g. prehospital transfusion, thoracotomy and resuscitative endovascular ballon occlusion of the aorta [REBOA]). Three independent reviewers systematically assessed each case to determine whether a potentially reversible cause of death was present and if advanced emergency interventions might have been applicable.

**Results:**

A total of 243 paediatric cases were included. The majority of deaths occurred prehospitally (91.6%). While the cause of death showed a significant association with age group (*p* = 0.01), severe traumatic brain injury (TBI) was present across all age groups without significant variation. Cardiopulmonary resuscitation was performed in 68.5% of cases, achieving return of spontaneous circulation only in 12.1%. Advanced emergency procedures were deemed feasible in only two cases (0.8%), including thoracotomy (*n* = 2), REBOA (*n* = 1), and prehospital transfusion (*n* = 2).

**Conclusion:**

This study reveals age-dependent patterns in paediatric trauma mortality and a very limited but relevant potential for advanced interventions. A rare subset, adolescents without severe TBI, might have benefited. Findings support structured trauma systems, targeted training, and selected use of prehospital transfusion.

**Trial registration:**

Not applicable.

**Supplementary Information:**

The online version contains supplementary material available at 10.1186/s12873-025-01469-5.

## Background

Trauma and its consequences are the leading cause of death among people under 40 years of age in Europe. Up to 66% of all trauma-related deaths occur in the prehospital setting, meaning that these patients never reach the emergency room. In 2021, the resulting accident-related costs for severely injured and deceased individuals amounted to €7.13 billion and €3.29 billion, respectively [1]. In the same year, 28,580 individuals were admitted to German hospitals who were either critically injured or died shortly after hospital admission. According to the German Trauma Registry, children account for 3.5% of these patients [2]. However, patients who died prior to hospital admission were not even included in these statistics.

In the first hour following trauma, the leading causes of death are exsanguination and severe traumatic brain injury (TBI). Post-mortem studies have shown that a significant proportion of preventable causes of death were either overlooked or not treated. It still remains unclear, how many trauma-related deaths can be considered potentially or definitively preventable [3, 4]. Forensic medical examinations play a crucial role not only in determining the cause of death, but also in identifying potentially reversible causes of cardiac arrest. This is of particular relevance, as most trauma registries exclude patients who die prior to hospital admission, thereby limiting the understanding of circumstances and characteristics associated with these fatalities. Several forensic studies have focused on trauma-associated fatalities; however, they have not specifically examined potentially reversible causes of death in children [3–8].

Potentially reversible causes of death are defined as those that could be prevented by emergency medical interventions. In children these can include basic measures such as airway management, but also advanced interventions like prehospital blood transfusions or invasive haemorrhage control techniques (e.g., emergency thoracotomy) [9–11]. Such interventions are already recommended by the European resuscitation guidelines, however, but mostly if only very limited experience is present [12]. It remains unclear whether and to what extent children with cardiac arrest may benefit from advanced interventions. Consequently, there is also uncertainty about the appropriate allocation of materials and expertise for this age group.

The primary objective of this study is to evaluate forensic post-mortem examinations to determine the proportion of cases in which advanced emergency interventions might have been applicable for the treatment of a potentially reversible cause of death.

## Methods

### Study design and ethics

This study presents a retrospective, single-centre study and was approved by the Ethics Committee of Justus Liebig University Giessen (Giessen, Germany; approval number AZ 200/23). The study was conducted in accordance with the principles of the Declaration of Helsinki [13]. Methods and results are reported in line with the Strengthening the Reporting of Observational Studies in Epidemiology (STROBE) guidelines [14]. Data were collected for all patients aged < 18 years who were examined post-mortem at the forensic department of the University Hospital of Giessen between January 1, 2011, and December 31, 2022.

The Institute of Legal Medicine in Giessen is responsible for the districts of the regional courts (Landgerichte) of Giessen, Limburg, Fulda, Marburg, and Kassel. Together, these regions cover a population of approximately 1.7 million people, making the cohort broadly representative of the German population. With the exception of Kassel (approx. 205,000 inhabitants), none of the district capitals are classified as large cities (> 100,000 inhabitants), reflecting a mix of urban and semi-urban areas. The main cities in the other court districts (Giessen, Marburg, Fulda, and Limburg) each have between 35,000 and 90,000 inhabitants, representing medium-sized urban centers typical for many parts of Germany.

### Data acquisition and definition of study parameters

Patients were identified manually by screening forensic records. All paediatric deaths were screened initially to avoid misclassification. Traumatic injuries, particularly in infants and in cases of non-accidental injury, may not be apparent from prehospital documentation or the initial emergency report. Forensic literature shows that trauma can be concealed behind presumed natural or unexplained causes (e.g., sudden infant death syndrome [SIDS]), and a complete case review was therefore required to reliably identify traumatic and potentially traumatic mechanisms [15, 16]. In addition, several advanced emergency procedures assessed in this study (e.g., emergency front-of-neck access, thoracic decompression, airway interventions) may also be relevant in non-traumatic causes of cardiac arrest such as airway obstruction or anaphylaxis. Stillbirths will be excluded.

Forensic files were screened and evaluated by three reviewers. The first reviewer is a forensic expert, while the second reviewer displayed a senior consultant for anaesthesiology and emergency medicine. If the reviews were not compatible, a third reviewer was asked for his opinion. This reviewer was also a senior anaesthetist and emergency physician. As part of the structured case analysis, the following factors were systematically evaluated: the presence of delays in emergency alerting or response times; missed injuries or incorrect diagnoses; the effectiveness of the emergency interventions performed; complications resulting from these interventions; omission of indicated procedures; and the extent to which the duration of the emergency mission was prolonged due to the measures undertaken.

The primary study aim is to evaluate forensic post-mortem examinations to identify the proportion of cases in which advanced emergency interventions might have been applicable for the treatment of a potentially reversible cause of death. Potentially reversible causes of death were identified based on established criteria from previous publications focusing on preventability of trauma-related deaths [5, 17]. The cause of death itself was determined based on autopsy findings and classified according to the dominant fatal injury mechanism. Injuries considered non-survivable included extensive rupture of the heart or major thoracic vessels, devastating brain injuries, brainstem herniation, dislocation of the upper cervical spine (C1–C3) or cranio-cervical junction with associated spinal cord injury, complete tracheal rupture, full-body burns or caustic injuries, and traumatic disruption or dismemberment of large body segments. Trauma-related deaths were defined as cases classified as polytrauma or severe isolated traumatic brain injury. All deaths attributed to pericardial tamponade, (tension) pneumothorax, hypoxia, and hypovolemia were analysed separately with regard to their traumatic or non-traumatic origin.

In contrast, conditions deemed potentially survivable, if recognized and treated with timely and appropriate emergency procedures, included haemothorax without massive cardiac or vascular rupture, tension pneumothorax, pericardial tamponade, exsanguination, hypoxia, failed airway management, and unrecognized intrathoracic or intra-abdominal injuries. Corresponding interventions may include prehospital blood transfusion, chest drainage, resuscitative thoracotomy, pericardiocentesis, REBOA, emergency amputation, advanced airway management (including video laryngoscopy or emergency front of neck access), and the use of diagnostic tools such as ultrasound.

### Statistical analysis

A descriptive statistical analysis was performed. Categorical data are reported as absolute and relative frequencies, while numerical variables are presented as medians with interquartile ranges (IQR).

To enhance transparency, the patient selection process is presented in a PRISMA-style flow diagram, detailing the application of inclusion and exclusion criteria.

Transportation times were estimated using Google Maps by determining the location of the event and calculating the ground transport distance in kilometres. Air transport times were estimated in minutes based on the direct flight path.

Determination of statistically significant association between two categorical variables has been tested by Fishers Exact test. A p-value ≤ 0.05 was considered statistically significant.

All statistical analyses were performed using R statistical software, version 4.2.3 (2023-03-15 ucrt) (www.r-project.org).

## Results

### Patient characteristics

**Fig. 1:**
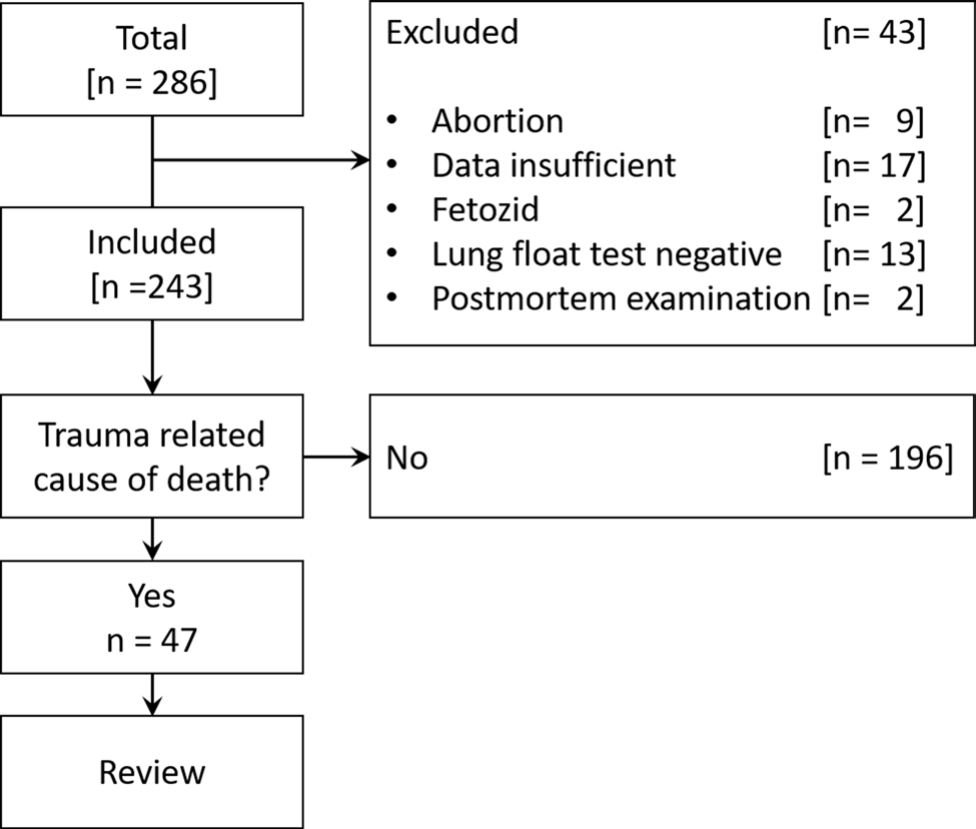
Flow diagram in PRISMA style showing case inclusion and exclusion throughout the study selection process

In total, 286 children and adolescents died during the study period of which 243 cases were included to the study (Fig. 1). In 97 cases (39.9%) the deceased was 6 years or older. The age at the time of death and other baseline characteristics are shown in Table 1. Over the study period, the number of cases varied to a little extent without significant deviations (median 19.5 [15.8–22.8] cases per year; minimum [2014, 2018, 2020]: 15 cases each [6.2%], maximum [2022]: 31 [12.8%] cases; Supplemental Fig. 1).

**Table 1:** Demographic data and basic characteristics

Parameter	Value
**Included cases according to age class (*n*** **[%])**	
Newborn (0–1 months)	36 (14.8)
Infant (2–12 months)	60 (24.7)
Toddler (12 months to < 6 years)	50 (20.6)
Schoolchild (≥ 6–<16 years)	32 (13.2)
Adolescents (≥ 12– <18 years)	65 (27.7)
**Age at time of death (mean ± SD)**	
Newborn (0–1 months, [months])	0.31 ± 0.47
Infant (2–12 months, [months])	5.13 ± 2.74
Toddler (12 months to < 6 years, [months])	31.28 ± 13.77
Schoolchild (≥ 6–<12 years, [years])	7.62 ± 2.12
Adolescents (≥ 12– <18 years, [years])	15.76 ± 1.53
**Sex**	
Male (n [%])	152 (62.6)
**Relevant pre-existing diseases (n (%))**	
None Neuropaediatric Oncologic Respiratory Metabolic disorders Genetic disorders (not other classified) Psychiatric disorders Cardiovascular disease Other	170 (70.0) 11 (4.5) 3 (1.2) 7 (2.9) 2 (0.8) 10 (4.1) 7 (2.9) 11 (4.5) 22 (9.1)

Abbreviation: SD = standard deviation

**Table 2:** Major injury regions, injury mechanisms, intent, and autopsy-based causes of death among polytrauma cases (*n* = 31)

Characteristics of patients with polytrauma (*n* = 31)	*n* (%)
**Location**	
Severe TBI	26 (83.9)
Thoracic	28 (90.3)
Abdominal Pelvis	14 (45.2) 8 (25.8)
Extremities	10 (32.3)
**Mechanism of injury**	
Blunt Pedestrian-vehicle collision Incident with traffic vehicle Incident with train Incident with airplane Fall from great height Blunt force trauma by assault	27 (87.1) 6 (19.4) 5 (16.1) 3 (9.7) 4 (12.9) 7 (22.5) 2 (6.5)
Penetrating Gunshot Stabbing	4 (12.9) 2 (6.5) 2 (6.5)
**Intent of injury**	
Homicide Suicide Accident	6 (19.4) 5 (16.1) 20 (64.5)
**Cause of death**	
Severe TBI Exsanguination Pericardial tamponade Haemato-/pneumothorax Body disruption	26 (83.9) 24 (77.4) 1 (3.2) 2 (6.5) 4 (12.9)

Abbreviations: TBI = traumatic brain injury

### Causes of death

Most deaths occurred in the prehospital setting (*n* = 222 [91.6%]), followed by the intensive care unit ([ICU], *n* = 7 [2.9%]). Of the in-hospital deaths, seven occurred in the intensive care unit ([ICU], [2.9%]) and two (0.8%) on the regular ward. Perioperative death was reported in 5 cases (2.1%).

**Fig. 2:**
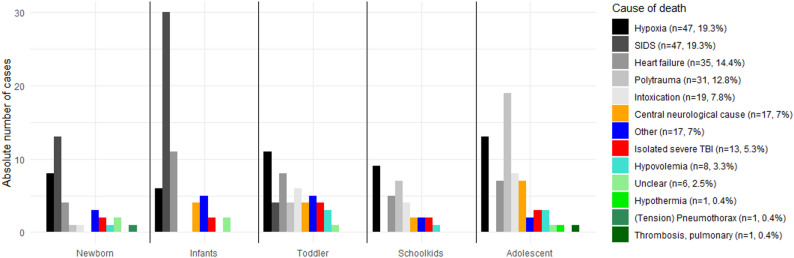
Causes of death according to age category. Trauma-related deaths included polytrauma and isolated severe traumatic brain injury (TBI). The single case of (tension) pneumothorax was classified as non-traumatic. Detailed causes of death among polytraumatized patients are presented in Table 2; Fig. 3. Abbreviation: SIDS = Sudden Infant Death Syndrome

The causes of deaths are shown in Fig. 2. Causes of death were statistically significant associated to different age classes (*p* = 0.01, Fig. 2). While newborn and infants were most likely to die from SIDS (newborn: *n* = 13 [36.1%], infant: *n* = 30 [50.0%]) toddler were most likely to die from hypoxia (*n* = 11, 22.1%). Schoolkids predominantly died from hypoxia (*n* = 14, 43.8%). The main predominant injury pattern leading to death in adolescents was polytrauma (*n* = 19, 29.2%), while hypoxia and intoxication (both *n* = 6, 9.2%) were equally common in second place in this age group.

Isolated severe TBI was present in all age categories without significant differences (Fig. 2) and accounted for 5.3% of all cases and 27.7% of all trauma-related deaths. In these cases, only minor non-lethal concomitant injuries were identified, and severe TBI was determined to be the primary cause of death.

**Fig. 3:**
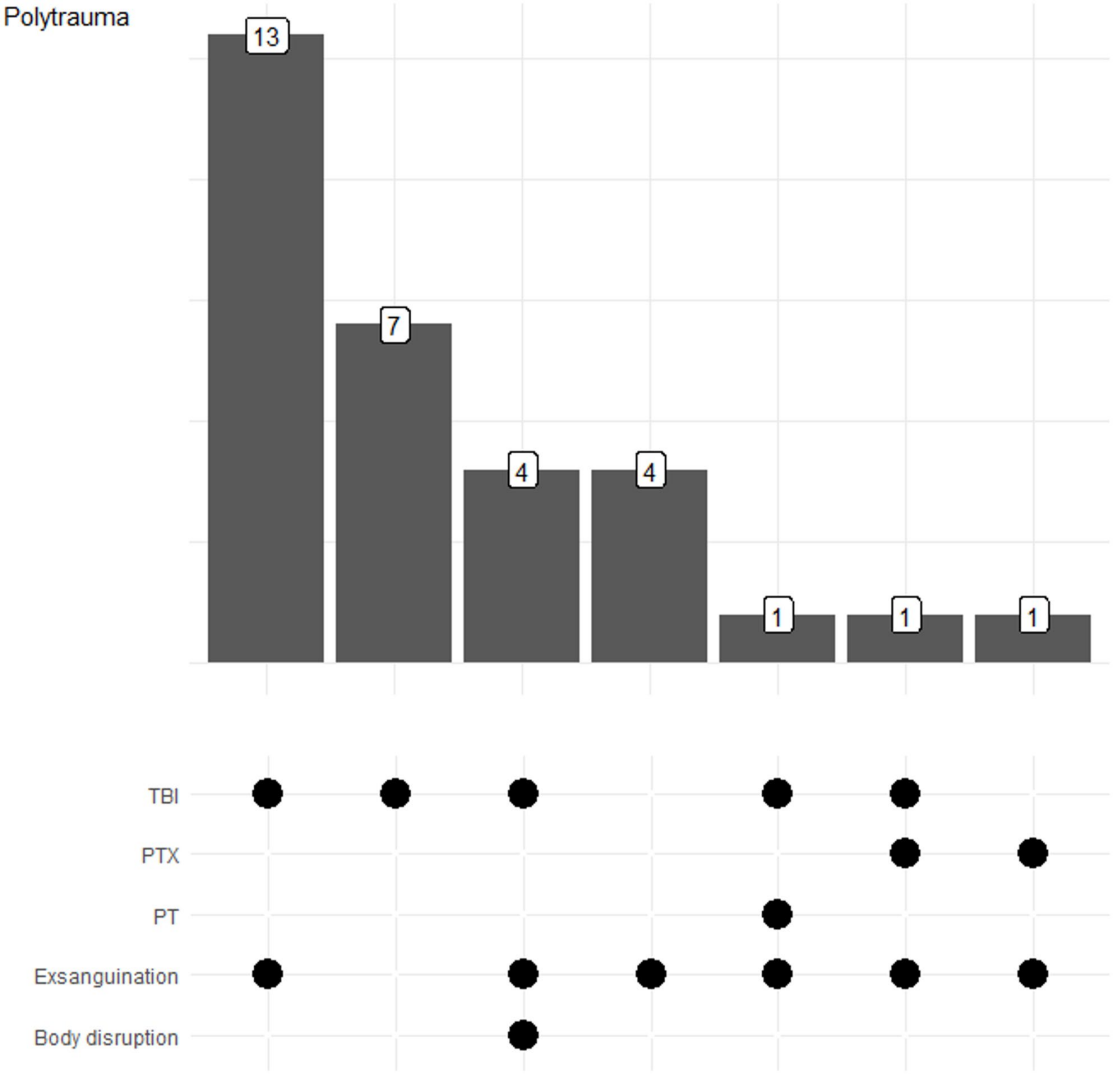
Causes of death in polytraumatized patients. Figure illustrating the distribution and overlap of fatal injury mechanisms in polytraumatized patients, as determined by autopsy findings, highlighting combinations of causes of death. Abbreviations: PT = pericardial tamponade; PTX = pneumothorax; TBI = traumatic brain injury

Severe TBI was present in 83.9% of all patients who died from polytrauma and represented the most frequent fatal injury in these patients (Table 2; Fig. 3), followed by exsanguination, which occurred in 77.4% of these patients. In 61.3% of polytraumatized patients, both exsanguination and severe TBI were present. Pericardial tamponade (*n* = 1) and tension pneumothorax (*n* = 2) were rare and were accompanied by either exsanguination or severe TBI (Fig. 3).

### Characterization of the performed emergency procedures

In total, 166 (68.5%) of cases cardiopulmonary resuscitation (CPR) was performed, of which 23 (12.1%) gained a return of spontaneous circulation (ROSC). In 76 (31.4%) of cases, no resuscitation was attempted.

Resuscitative interventions included advanced airway management with endotracheal intubation in 81 (42.6%) of cases and insertion of a laryngeal mask in 9 cases (4.7%). Defibrillation was rarely needed (2 [1.1%]). Intraosseous access was performed in 40 (21.1%), while peripheral venous access in 74 (38.9) and central venous access in 44 (21.1%) cases. Decompression of pneumo- or haemothorax was performed in 10 (5,3%) of cases.

In cases of traumatic cardiac arrest (defined as hypovolemia, (tension-)pneumothorax, cardiac tamponade and polytrauma) the decompression of the thorax was performed in 4 of 10 (40%) cases. Overall, thoracic decompression by thoracocentesis was performed in all age classes except the group of toddlers (newborn: 2/36; infants: 1/60; schoolkids: 2/32; adolescents 5/65).

Diagnostic procedures, which would also be available in the prehospital setting, included sonography in 88 (36.2%) and blood gas analysis also in 88 (36.2%) of cases.

### Evaluation of the potential of invasive procedures

In total, the potential for invasive procedures was assessed in 47 patients. Most reviews were conducted in patients older than 5 years, with 28.1% of reviewed cases involving school-aged children and 41.5% adolescents. In only three cases did the reviewers disagree on whether the injuries were survivable. In these instances, a third reviewer was consulted; the final judgment was that one of the injuries were treatable, and two were not. Supplemental Table 1 provides an overview of all discussed procedures, including the three cases with divergent reviewer opinions (*Note to the editor*: Table 2*could be shown here*).

**Fig. 4:**
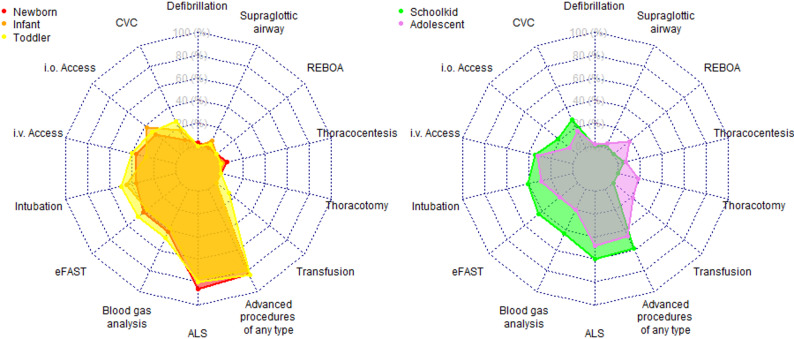
Spider web graph demonstrating different spectrum of necessary procedures depending on age class. Results are given as proportion of all respective cases. Abbreviations: ALS = advanced life support; CVC = central venous catheterization; eFAST = emergency Focused Assessment with Sonography for Trauma; i.v. = intravenous; i.o. = intraosseous; REBOA = Resuscitative Endovascular Balloon Occlusion of the Aorta

Overall, advanced resuscitative procedures were considered feasible in two cases (0.8% of all included patients and 4.2% of patients included to the review) according to the reviewers (Supplemental Table 1, Fig. 4). These included thoracotomy in both cases, REBOA in one case, and prehospital blood transfusion in both cases. Both patients were adolescents (14 years and 10 months; 15 and 6 months).

To evaluate the practicality of invasive procedures, the location of the emergency relative to the nearest hospital was analysed. In 41 cases of traumatic cardiac arrest, the mean distance to a tertiary trauma centre was 12.8 ± 5.01 km, with a median (IQR) of 16 [8–17] km. This corresponded to a mean ground transport time of 23.3 ± 12.5 min and a mean air transport time of 4.5 ± 2.9 min. One-quarter of patients (49 cases, 25.8%) had an estimated ground transport time exceeding 30 min, while only one patient (0.5%) had an estimated air transport time longer than 15 min. Considering the transport durations, advanced resuscitative procedures were deemed indicated in all cases. In one instance, ground transport time exceeded 60 min, underscoring the necessity for helicopter emergency medical service (HEMS).

## Discussion

This study aimed to identify paediatric trauma cases in which advanced emergency procedures might have been indicated, using a representative cohort from a Western European setting. Most deaths occurred in the prehospital setting, particularly among newborns and infants, where SIDS was the most frequent cause. In contrast, polytrauma was the leading injury pattern in adolescents, while hypoxia, as a primary or secondary mechanism, was prevalent across all age groups. These results are in line with a recent analysis of the German Resuscitation Registry [18]. The majority of children in trauma-related cardiac arrest were first managed by EMS. Despite a high rate of CPR attempts (68.5%), ROSC was achieved in only 12.1% of cases, underscoring the severity and frequently non-survivable nature of these emergencies. While common interventions such as airway management, vascular access, and thoracic decompression were performed, advanced procedures like resuscitative thoracotomy or REBOA were not attempted.

Only 0.8% of patients were retrospectively judged as potentially eligible for such interventions, all of whom were adolescents. In younger children, advanced procedures were deemed non-beneficial, primarily due to the prevalence of SIDS and severe TBI. TBI was a major contributing factor across all age groups and is known to be one of the leading causes of paediatric trauma mortality. In addition to isolated TBI, which accounted for 27.7% of all trauma-related deaths, severe TBI was frequently present in polytraumatized patients (83.9%) and was commonly accompanied by exsanguination (61.3%). Severe TBI is associated with a particularly poor prognosis, with reported mortality rates ranging from 20% to over 50% depending on the extent of primary injury, secondary insults, and access to timely neurosurgical care [19]. In our cohort, severe TBI was present across all age categories, consistent with its role as a cross-cutting mechanism of fatal injury in paediatric patients. Although the frequency did not differ significantly between age groups, its presence as an underlying or contributing cause of death underscores the importance of rapid recognition and aggressive management, including early airway protection, prevention of secondary brain injury, and timely neurosurgical evaluation when appropriate [20]. However, invasive procedures usually do not improve the survival of TBI and hypoxia and might explain therefore the little number of indications in the cohort of infants, preschool and school children. In the few eligible cases, absence of TBI, adolescent age, and injury mechanisms (e.g., exsanguinating haemorrhage or thoracic trauma) made advanced interventions theoretically feasible. REBOA, for example, was only applicable in older patients due to anatomical constraints and the lack of paediatric-specific systems. This is reasoned by the fact that no paediatric REBOA systems are available making a 6–12 F catheter sheath necessary [21]. Supporting this, a recent study by Theodorou et al. suggested that nearly 20% of severely injured children could theoretically benefit from REBOA, highlighting the need for further device development and research [22].

Emergency thoracotomy was similarly considered feasible only in older children and adolescents and, even then, only theoretically feasible in two patients. Overall, classical indications for resuscitative thoracotomy in children, such as pericardial tamponade or tension pneumothorax, were rare, possibly reflecting the greater flexibility of the paediatric thoracic cage. This is consistent with the largest systematic review on paediatric emergency thoracotomy, which included 252 cases with a median age of 15 years. Survival was highly dependent on the mechanism of injury: 1.6% for blunt trauma and 10.2% for penetrating trauma. Among children under 12 years, only one survivor was reported, a 9-year-old with a cardiac stab wound [23]. The recent landmark study by Perkins et al., based on data from the London HEMS, provides no information on children receiving prehospital resuscitative thoracotomy [24]. However, it emphasizes that this procedure is only effective when performed by experts within a narrow time window after the onset of cardiac arrest, and preferably in patients with cardiac tamponade. In our cohort, this condition was present in only one child, who also suffered from severe TBI.

The limited applicability of emergency thoracotomy in children can be explained using the “4 Es” framework (Expertise, Equipment, Elapsed time, and Environment), as proposed in European trauma guidelines [25]. First, there is no standardized definition of expertise for this procedure, and formal paediatric training programs are lacking. Second, specialized equipment is rarely available in prehospital settings, and adult tools may not be suitable for paediatric anatomy. Third, while no paediatric-specific timeframes exist, the adult-based cut-off of 10–15 min without signs of life appears reasonable. Finally, such procedures require a trauma system with paediatric thoracic and cardiac surgical capabilities, as well as access to blood products, particularly important given that many eligible patients in this study had estimated transport times exceeding 30 min.

Given these constraints, prehospital transfusion appears to be the only realistically applicable advanced intervention across all paediatric age groups. A recent study by Morgan et al. involving 559 children showed that early transfusion at the scene significantly reduced 24-hour and in-hospital mortality compared to transfusion initiated in hospital [26]. Given its potential relevance and feasibility, future research should focus on evaluating the impact of prehospital blood transfusion, including whole blood strategies, on survival and early resuscitation outcomes in paediatric trauma.

From a systems perspective, the extremely low number of potentially preventable deaths identified in our cohort (two cases over an eleven-year period) has important implications for feasibility and cost–benefit. Advanced invasive procedures such as resuscitative thoracotomy or REBOA are highly complex, require substantial training and maintenance of procedural competence, and are associated with relevant risks and resource use. Given the very small absolute number of eligible paediatric trauma patients, it is unlikely that paediatric-specific prehospital programmes for such procedures could be organised in a cost-effective manner. Existing data on REBOA stem almost exclusively from adult populations and remain inconclusive with regard to a clear survival benefit and cost-effectiveness even in high-volume settings [27, 28]. In contrast, prehospital transfusion of blood products represents a more broadly applicable intervention that may benefit both adults and children at risk of exsanguination. Adult economic evaluations of prehospital plasma programmes suggest that such strategies can be cost-effective in selected systems, particularly when integrated into existing air medical services and targeted to patients with a high a priori risk of haemorrhagic shock, although the overall evidence remains mixed and largely observational [29, 30]. Paediatric data are limited to registry and cohort studies, which indicate feasibility and a potential association with improved outcomes, but do not allow firm conclusions about cost–effectiveness.

Rather than advocating for stand-alone paediatric trauma systems with dedicated high-complexity procedures, our findings support the concept of embedding paediatric expertise and needs into existing trauma networks and advanced retrieval systems that predominantly serve adult patients. Within such structures, generalizable interventions with comparatively favourable risk–benefit profiles, such as rapid haemorrhage control, timely transport to specialised centres, and, where available, prehospital transfusion of blood products, are likely to provide the greatest value for all trauma patients.

However, this study is subject to several limitations inherent to retrospective designs. First, missing data and incomplete documentation may have led to underreporting of certain procedures. Second, the retrospective assessment of the potential benefit of invasive procedures involves subjective judgment, despite the use of multiple independent reviewers. Third, the absence of a control group or surviving cohort limits conclusions regarding the actual effectiveness of interventions.

## Conclusion

This retrospective study highlights age-specific patterns in paediatric trauma-related mortality and the limited but relevant potential of advanced emergency interventions. While most cases were non-survivable or accompanied by severe TBI, a small subset, primarily adolescents with traumatic cardiac arrest without severe TBI, might have benefited from timely advanced procedures. Ideally, these measures should be complemented by prehospital transfusion, administered to the right patient, at the right time, and for the right indication. These findings underscore the need for structured paediatric trauma systems, improved training, and prehospital capabilities tailored to children and adolescents.

## Supplementary Information

Below is the link to the electronic supplementary material.

Supplementary Material 1

Supplementary Material 2: Supplemental Fig. 1. Development of deceased patients over the study period

## Data Availability

The datasets used and/or analysed during the current study are available from the corresponding author on reasonable request.

## Abbreviations

ALS: Advanced life support
CVC: Central venous catheterization
eFAST: Emergency focused assessment with sonography for trauma
i.o.: Intraosseous
i.v.: Intravenous
IQR: Interquartile range
HEMS: Helicopter emergency medical system
REBOA: Resuscitative endovascular ballon occlusion of the aorta
RCX: Ramus circumflexus
ROSC: Return of spontaneous circulation
STROBE: Strengthening the Reporting of Observational Studies in Epidemiology
SIDS: Sudden Infant Death Syndrome
TBI: Traumatic brain injury
