# Supplementary material for: Reversible causes of death and the potential benefit of invasive emergency techniques in paediatric and adolescent trauma: a 12-years retrospective forensic analysis
Source: BMC Emerg Med. 2026 Jan 9;26:29. doi: 10.1186/s12873-025-01469-5 (PMC12849071; doi:10.1186/s12873-025-01469-5)
Supplement: Supplementary file 1 — Supplementary Material 1 [file 12873_2025_1469_MOESM1_ESM.docx]

**Supplemental Table 1.** Characterization of the cases with potential interventions.

| **Number** | **Age** | **Mechanism of injury** | **Forensic presentation/injury pattern** | **Review consensus/**  **Reason** | **Potential advanced intervention** | **Time to Transport / Airline Distance** |
| --- | --- | --- | --- | --- | --- | --- |
| 1 | 19 months | Fatal blunt trauma | TBI with subarachnoid haemorrhage, fracture of the first cervical vertebra, skull base fractures | Untreatable  Reason: severe TBI | none | Ground 19 min  Air Line 12 km |
| 2 | 17 years and 8 months | Homicide | Polytrauma, A total of 7 gunshot wounds, through-and-through injury of the abdominal aorta, additional entry wound in the left thorax with left-sided pneumothorax, liver laceration, diaphragmatic injury. | ***Third reviewer decision:***  2 untreatable/1 treatable  Decision: untreatable  Reason: unrepairable injury of the aorta | none | Ground 8 min  Air Line 2 km |
| 3 | 16 years and 3 months | Train accident (suicide) | Decapitation | Untreatable  Reason: unsurvivable TBI | none | Ground 52 min  Airline 37 km |
| 4 | 5 years and 9 months | Bicycle–car collision | TBI with cerebral oedema | Untreatable  Reason: unsurvivable TBI | none | Ground 25 min  Airline 22 km |
| 5 | 17 years and 10 months | Homicide–suicide | Gunshot wound to the head with subsequent burning in the vehicle | Untreatable  Reason: unsurvivable TBI/burning | none | Ground 22 min  Airline 18 km |
| 6 | 16 years and 4 months | Run-over injury by motor vehicle | Polytrauma, heart and lungs impaled by ribs, multiple pelvic fractures | Untreatable  Reason: unsurvivable heart injuries | none | Ground 43 min  Airline 26 km |
| 7 | 15 years and 1 month | Train accident (suicide) | Polytrauma, brain exenteration, multiple rib and pelvic fractures | Untreatable  Reason: unsurvivable TBI | none | Ground 22 min  Airline 15 km |
| 8 | 17 years and 9 months | Pedestrian–vehicle collision | Polytrauma, Severe TBI, intraventricular haemorrhage, vascular injuries of the basal ganglia, head and neck avulsion with lacerations, haematopneumothorax on the right sight (1,009 mL), right renal vein avulsed at the lower pole | untreatable  Reason: Failure of PALS because of severe TBI | none | Ground 12 min  Airline 8 km |
| 9 | 16 years and 5 months | Passenger ejected from vehicle in motor vehicle collision | Polytrauma, Open arterial injury of the left upper arm, left-sided predominant lung injury/contusion, pulmonary artery avulsion and bronchial rupture, liver fragmentation (or severe liver laceration), liver vein avulsion at the inferior vena cava, liver tissue flushed into the pulmonary arteries | Untreatable  Reason: unsurvivable injury of the pulmonary artery | none | Ground 20 min  Airline 11 km |
| 10 | 15 years and 5 months | Train accident (suicide) | Polytrauma, comminuted fracture of the skull, brain exenteration, amputation of right hand and both feet, left thoracic trauma, lung contusion, bilateral haemothorax | Untreatable  Reason: unsurvivable TBI | none | Ground 15 min  Airline 8 km |
| 11 | 6 years and 4 months | Plane crash | Polytrauma, completely shattered skull, flail chest, multiple fractures of all extremities | Untreatable  Reason: unsurvivable TBI | none | Ground 26 min  Airline 22 km |
| 12 | 8 years and 6 months | Plane crash | Polytrauma, completely destroyed skull, brain exenteration, flail chest, complete rupture of the heart, avulsion of the thoracic artery, multiple fractures of the upper and left lower extremities | Untreatable  Reason: unsurvivable TBI | none | Ground 26 min  Airline 22 km |
| 13 | 2 years and 10 months | Plane crash | Polytrauma, complete comminution of the head, severe thoracic, abdominal, and extremity trauma with multiple fractures, rupture of heart, liver, and spleen. | Untreatable  Reason: unsurvivable TBI | none | Ground 26 min  Airline 22 km |
| 14 | 4 years and 5 months | Plane crash | Polytrauma, complete comminution of the head, severe thoracic, abdominal, and extremity trauma with multiple fractures, rupture of heart, liver, and spleen. | Untreatable  Reason: unsurvivable TBI | none | Ground 26 min  Airline 22 km |
| 15 | 2 years and 5 months | Child abuse, blunt force trauma to the head | Polytrauma, Severe TBI with cerebral oedema, multiple injuries to the pelvis, knees, legs, and feet | Untreatable  Reason: unsurvivable TBI | none | Ground 42 min  Airline 29 km |
| 16 | 1 months | Child abuse | Polytrauma, Serial rib fractures, cardiac contusion, lung contusion, tear of the right atrium, injury to the left anterior wall of the heart, left hemothorax, liver laceration | Untreatable  Reason: unsurvivable heart injury | none | Ground 22 min  Airline 17 km |
| 17 | 15 years and 7 months | Highway pedestrian collision with secondary run-over | Polytrauma, severe TBI with comminuted fractures, fracture of the second cervical vertebra, bilateral serial rib fractures | Untreatable  Reason: unsurvivable TBI | none | Ground 11 min  Airline 4 km |
| 18 | 4 months | Shaken baby syndrome | Bony skull injuries, subarachnoid haemorrhage, bilateral frontal bone fractures, subdural hematoma. | Untreatable  Reason: unsurvivable TBI | none | Ground 52 min  Airline 37 km |
| 19 | 16 years and 4 months | Drowning after traffic accident  (car into lake) | Recovered after 2 hours of search, lung contusion | Untreatable  Reason: prolonged drowning | none | Ground 24 min  Airline 21 km |
| 20 | 10 months | Shaken baby syndrome | TBI with central autonomic regulatory failure | Untreatable  Reason: unsurvivable TBI | none | Ground 17 min  Airline 9 km |
| 21 | 15 years and 6 months | Pedestrian–vehicle collision | Polytrauma, Massive thoracic trauma with rib fractures, impalement injury of the right lung, 300 ml blood in the right thorax, spleen laceration, liver laceration, left-sided skull base fracture extending completely from the posterior cranial fossa via the sella turcica to the anterior cranial fossa. | Untreatable  Reason: unsurvivable TBI | none | Ground 46 min  Airline 33 km |
| 22 | 16 years and 8 months | Train accident (suicide) | Decapitation at the level of the skull base, body subjected to traction | Untreatable  Reason: unsurvivable TBI | none | Ground 13 min  Airline 6 km |
| 23 | 16 years and 7 months | Passenger ejected from vehicle in motor vehicle collision | Polytrauma, Severe TBI with rupture of the ligamentous structures of the cranio-cervical junction, brainstem contusion, lung impalement, liver and spleen lacerations, anterior skull base fracture, serial rib fractures, pubic bone fractures, extremity fractures. | Untreatable  Reason: unsurvivable TBI | none | Ground 22 min  Airline 16 km |
| 24 | 14 years and 10 months | Pedestrian run over twice by a vehicle | Polytrauma, Multiple internal injuries, spleen and liver rupture, rib fractures, pelvic fractures, collapse of the left lung, haemothorax. | ***Third reviewer decision:***  1 untreatable/2 treatable  Decision: treatable  Reason: lack of severe TBI and potentially treatable bleeding locations | REBOA,  Resuscitative thoracotomy,  Prehospital blood transfusion | Ground 17 min  Airline 8 km |
| 25 | 9 years and 2 months | Homicide–suicide (Stab wound to the chest followed by a fall from 95 meters from a bridge) | Polytrauma, stab wound to the left upper torso, skull suture diastasis, fractures of the cranial and facial bones, fracture of the third cervical vertebra (C3), pericardial rupture, liver and spleen lacerations, gastrointestinal tract tears. | Untreatable  Reason: unsurvivable TBI | none | Ground 18 min  Airline 15 km |
| 26 | 10 years and 2 months | Homicide–suicide  (Stab wound to the chest followed by a fall from 95 meters from a bridge) | Polytrauma, stab wound to the apex of the right heart, multiple skull fractures, avulsion of the medulla oblongata, multiple vertebral fractures, sternum, rib, and pelvic fractures | Untreatable  Reason: unsurvivable TBI | none | Ground 18min  Airline 15km |
| 27 | 12 years and 4 months | Fall from approximately 9 meters height | Polytrauma, Pneumothorax, and multiple subarachnoid and intracerebral hemorrhages, fracture of the neurocranium with signs of brain herniation followed by brain death, left femoral shaft fracture, | Untreatable  Reason: unsurvivable TBI | none | Ground 48 min  Airline 39 km |
| 28 | 17 years and 9 months | Suicide by jump from height (47 m) | Polytrauma, shattered skull with shard-like comminution, impalement of the cervical spine into the skull, sternum fractures, bilateral serial rib fractures, right lung puncture, pericardial tear, avulsion of the descending aorta | Untreatable  Reason: unsurvivable TBI and aortic injury | none | Ground 28min  Airline 17km |
| 29 | 14 years and 8 months | Fall of great height (suicide) | Polytrauma, severe TBI, fracture line through both orbits, skull base hinge fracture, blood aspiration into both lungs, subdural haemorrhages, fracture of the left second rib, 1100 ml blood in the thoracic cavity, rupture of the vena cava, tears in the heart chambers | ***Third reviewer decision:***  2 untreatable/1 treatable  Decision: Untreatable  Reason: unsurvivable TBI and injuries of the great vessels | none | Ground 12min  Airline 3km |
| 30 | 17 years and 9 months | Fall from 20 meters height | Polytrauma, Severe TBI, blunt thoracic trauma, bilateral haemothorax, pericardial tear, tracheal rupture | Untreatable  Reason: unsurvivable TBI and tracheal rupture | none | Ground 23 min  Airline 11 km |
| 31 | 16 years and 10 months | Unclear severe trauma to the head | Severe TBI, subdural hematoma with signs of increased intracranial pressure, marked impressions of the right hemisphere | Untreatable  Reason: unsurvivable TBI | none | Ground 42 min  Airline 26 km |
| 32 | 15 years and 6 months | Homicide | Seven stab wounds, massive blood loss, perforation of the right pulmonary artery, small injury to the inferior vena cava at the level of the diaphragm | Treatable  Reason: Potentially reversible cause of death (compression of the right pulmonary artery) | Resuscitative thoracotomy,  Prehospital blood transfusion | Ground 62 min  Airline 46 km |
| 33 | 17 years and 3 months | Fall of great height | Polytrauma, comminuted fracture of the right cranial vault, fracture of the middle and posterior cranial fossae as well as the right petrous bone, contusion haemorrhages, right hemopneumothorax with 580 ml blood, contusion haemorrhages of both lungs, spleen rupture, rupture of the right kidney, capsular haemorrhages of the left kidney, multiple fractures of the pelvis and thorax | Untreatable  Reason: unsurvivable TBI | none | Ground 20 min  Airline 15 km |
| 34 | 5 years and 1 month | Traffic accident | Open TBI diagnosed on site, trapped between the front passenger seat, injury to the circle of Willis arteries. | Untreatable  Reason: unsurvivable TBI | none | Ground 21 min  Airline 18 km |
| 35 | 14 years and 7 months | Bus–pedestrian collision | Polytrauma, partial brain removal and avulsion of the brainstem in an open TBI, shard-like fractures of the facial skull, skull base, and cranial vault, multiple bilateral rib fractures, spinous process fractures of the thoracic spine, pelvic fracture with symphyseal disruption, multiple fractures of the upper and lower extremities, tear of the left carotid artery, avulsion of the trachea below the larynx, lacerations of the liver and lung | Untreatable  Reason: unsurvivable TBI | none | Ground 49 min  Airline 37 km |
| 36 | 1 month | Shaken baby syndrome | Severe TBI, Skull fractures, | untreatable Reason: unsurvivable TBI | none | Ground 4min  Airline 11km |
| 37 | 2 years and 7 months | Severe blunt trauma to the head | Isolated severe TBI, craniotomy, brain death | Untreatable  Reason: unsurvivable TBI | none | Ground 36min  Airline 25km |
| 38 | 9 years | Homicide with Gunshot wound to the head | Polytrauma, brain exenteration, extensive facial and cranial fractures, partial absence of the left skull hemisphere, multiple fractures of all extremities, aortic rupture in the arch, liver fragmentation, stomach and spleen lacerations. | Untreatable  Reason: unsurvivable TBI | none | Ground 34min  Airline 30km |
| 39 | 17 years and 9 months | Gunshot wound to the head | Gunshot wound to the head with transection of the brainstem | untreatable  Reason: unsurvivable TBI | none | Ground 25min  Airline 22km |
| 40 | 16 years and 9 months | Traffic accident | Polytrauma, unstable cervical spine fractures at C5–C7, torn thoracic spine ligaments at T10–T11, pelvic fracture, fractures of the anterior to posterior cranial fossae, traumatically opened chest cavity, sternum fracture, rib fractures ribs 2–4 right, 1–3 left, multiple liver lacerations, left kidney torn at the upper pole, transverse colon laceration, pericardial rupture, bilateral pleural impalements, deep laceration on the right | untreatable  Reason: unsurvivable chest injuries | none | Ground 26min  Airline 22km |
| 41 | 15 years and 11 months | High-velocity trauma during car accident | Open TBI | untreatable  Reason: unsurvivable TBI | none | Ground 34min  Airline 25km |
| 42 | 2 years and 5 months | Pedestrian–vehicle collision | Severe TBI with craniotomy, extensive brain contusion of the right telencephalon, fracture of the right posterior parietal bone. | Untreatable  Reason: unsurvivable TBI | none | Ground 21min  Airline 12km |
| 43 | 1 year and 4 months | Traffic accident with car occupant ejected | Polytrauma, subarachnoid haemorrhage, epidural haemorrhage, skull fractures, haemorrhages of the endocardium, thymus, splenic infarcts, pulmonary oedema | Untreatable  Reason: unsurvivable TBI | none | Ground 33min  Airline 33km |
| 44 | 14 years and 9 months | High-velocity trauma during car accident | Polytrauma, internal haemorrhage, severe TBI, skull base fracture involving the anterior and middle left cranial fossae, bilateral sacroiliac joint disruption, fractured right iliac wing, fractured right pubic bone, fractured right acetabulum. | untreatable  Reason: unsurvivable TBI | none | Ground 21 min  Airline 14 km |
| 45 | 13 years and 7 months | Suspected fall from great height | Polytrauma, severe TBI, skull base fracture, thoracic trauma, cardiac tamponade. | Untreatable  Reason: unsurvivable TBI and heart injury | none | Ground 5 min  Airline 1 km |
| 46 | 5 years and 2 months | Fall of great height | Polytrauma, fracture line in both middle cranial fossae, subdural and epidural hematomas, cortical and medullary haemorrhages, liver rupture, lung contusion with lung rupture | Untreatable  Reason: unsurvivable TBI and liver injury | none | Ground 8 min  Airline 1 km |
| 47 | 8 years and 2 months | Pedestrian-train accident | Polytrauma, TBI and torso trauma, tears at the root of the pulmonary artery as well as tears of the RCX | Untreatable  Reason: unsurvivable TBI and chest injury | none | Ground 23 min  Airline 10 km |

*Abbreviations: RCX = ramus circumflexus, REBOA = Resuscitative Endovascular Balloon Occlusion of the Aorta; TBI = Traumatic brain injury*
