# Supplementary figures and images for: Reversible causes of death and the potential benefit of invasive emergency techniques in paediatric and adolescent trauma: a 12-years retrospective forensic analysis
Source: BMC Emerg Med. 2026 Jan 9;26:29. doi: 10.1186/s12873-025-01469-5 (PMC12849071; doi:10.1186/s12873-025-01469-5)

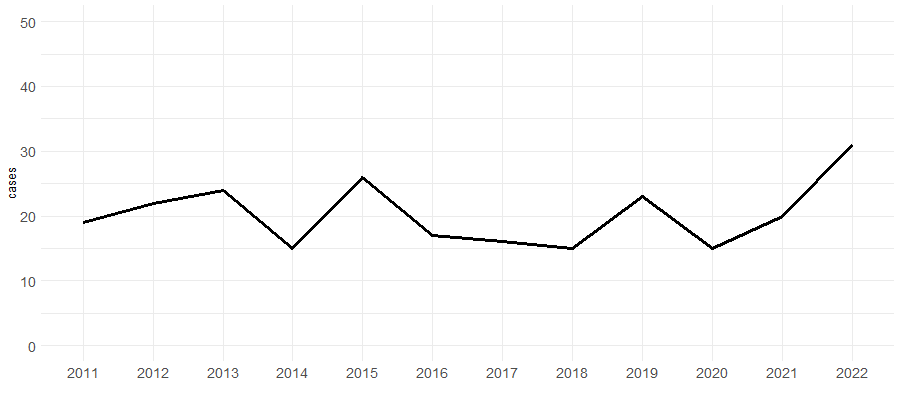

Supplement: Supplementary file 2 — Supplementary Material 2: Supplemental Fig. 1. Development of deceased patients over the study period [file 12873_2025_1469_MOESM2_ESM.tif]
